# Sesquiterpenes and Cyclodepsipeptides from Marine-Derived Fungus *Trichoderma longibrachiatum* and Their Antagonistic Activities against Soil-Borne Pathogens

**DOI:** 10.3390/md18030165

**Published:** 2020-03-16

**Authors:** Feng-Yu Du, Guang-Lin Ju, Lin Xiao, Yuan-Ming Zhou, Xia Wu

**Affiliations:** 1 College of Chemistry and Pharmacy, Qingdao Agricultural University, Qingdao 266109, China; fooddfy@126.com (F.-Y.D.); jgl2018666@163.com (G.-L.J.); xiaolin_qd@163.com (L.X.); 2 Shandong Key Laboratory of Applied Mycology, Qingdao Agricultural University, Qingdao 266109, China; 3 Analytical and Testing Center, Qingdao Agricultural University, Qingdao 266109, China; zym7410@163.com; 4 Key Lab of Integrated Crop Pest Management of Shandong Province, College of Plant Health and Medicine, Qingdao Agricultural University, Qingdao 266109, China

**Keywords:** marine-derived fungus, sesquiterpenes, cyclodepsipeptides, antagonistic activity

## Abstract

Soil-borne pathogens, including phytopathogenic fungi and root-knot nematodes, could synergistically invade vegetable roots and result in serious economic losses. The genus of *Trichoderma* has been proven to be a promising reservoir of biocontrol agents in agriculture. In this study, the search for antagonistic metabolites from a marine-derived fungus, *Trichoderma longibrachiatum,* obtained two structural series of sesquiterpenes **1**–**6** and cyclodepsipeptides **7**–**9**. Notably, the novel **1** was a rare norsesquiterpene characterized by an unprecedented tricyclic-6/5/5-[4.3.1.0^1,6^]-decane skeleton. Their structures were elucidated by extensive spectroscopic analyses, while the absolute configuration of novel **1** was determined by the comparison of experimental and calculated ECD spectra. The novel **1** and known **2** and **3** showed significant antifungal activities against *Colletotrichum lagrnarium* with MIC values of 8, 16, and 16 μg/mL respectively, even better than those of the commonly used synthetic fungicide carbendazim with 32 μg/mL. They also exhibited antifungal potential against carbendazim-resistant *Botrytis cinerea*. Cyclodepsipeptides **7**–**9** showed moderate nematicidal activities against the southern root-knot nematode (*Meloidogyne incognita*). This study constitutes the first report on the antagonistic effects of metabolites from *T. Longibrachiatum* against soil-borne pathogens, also highlighting the integrated antagonistic potential of marine-derived *T. Longibrachiatum* as a biocontrol agent.

## 1. Introduction

*Colletotrichum* spp., *Botrytis cinerea*, and *Fusarium oxysporum* are three well-known and seriously damaging soil-borne phytopathogenic fungi worldwide, which cause anthracnose, gray mold, and wilt diseases of vegetables, respectively [1]. The southern root-knot nematode (*Meloidogyne incognita*) is also a typical soil-borne pathogen which invades vegetable roots [2,3]. More seriously, soil-borne fungi could synergistically interact with southern root-knot nematodes and therefore result in an even greater threat to vegetable cultivation [4,5]. Hence, the development of integrated control agents is always in demand.

Currently, the main strategy for controlling soil-borne pathogens is highly dependent on synthetic agrochemicals [6]. However, their ongoing overuse has resulted in a series of negative consequences, such as residual toxicity, the developing resistance of targeted pathogens, and many other environmental issues [6,7]. Therefore, the search for environmentally friendly integrated control alternatives has attracted more and more attention. Notably, the genus of *Trichoderma* has been successfully applied to control soil-borne pathogens, such as *Trichoderma harzianum* and *T. viride* [8,9,10]. Recently, the species of *T. longibrachiatum* has also been suggested as a biocontrol agent against phytopathogens due to its potent antagonistic effect [11,12,13]. Research on its mechanism has mainly focused on its mycoparasitic and enzyme-producing abilities, with few reports on its antagonistic metabolites [12,13,14]. *T. longibrachiatum* is generally isolated from terrestrial soil and plants [11,12,13,14], being a rare marine resource [15]. Ji and co-workers isolated various *Trichoderma* species from marine alga which could produce structurally unique and biologically active metabolites [16,17,18,19], also indicating the research potential of marine-derived *Trichoderma* species.

**Figure 1:**
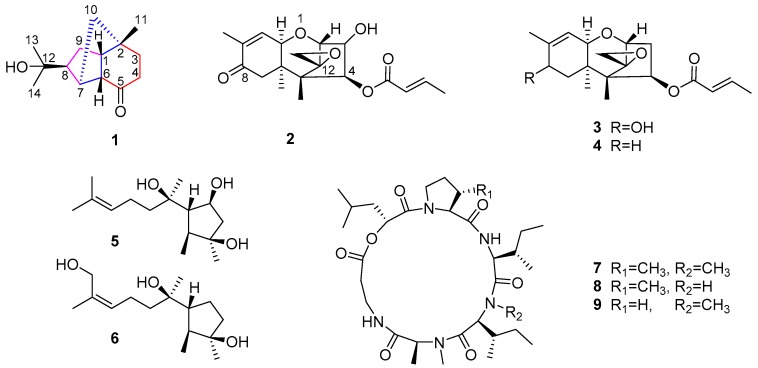
Structures of sesquiterpenes **1**–**6** and cyclodepsipeptides **7**–**9**.

During our ongoing search for biocontrol agents in agriculture [20,21,22], a marine-derived fungus *T. longibrachiatum* attracted our attention due to its potent antagonistic ability to destroy soil-borne pathogens. Furthermore, the search for its antagonistic metabolites obtained two structural series of sesquiterpenes **1**–**6** and cyclodepsipeptides **7**–**9** (Figure 1), especially including a new rare norsesquiterpene **1**. The isolation, structural elucidation, and antagonistic evaluation of the isolated metabolites (**1**–**9**) are discussed herein.

## 2. Results and Discussion

### 2.1. Structural Elucidation

The molecular formula of compound **1** was obtained as C_14_H_22_O_6_ by HRESIMS (Figure S1 in materials, SI), implying four degrees of unsaturation. The one-dimensional NMR and HSQC data (Table 1 and Figure S5) exhibited one carbonyl carbon (*δ*_C_ 214.7), one oxygenated (*δ*_C_ 73.6) and one aliphatic (*δ*_C_ 43.4) quaternary carbons, four CH (*δ*_C_ 66.1, 48.0, 37.5, and 37.1), four CH_2_ (*δ*_C_ 35.9, 33.0, 20.6, and 20.3) and three CH_3_ groups (*δ*_C_ 28.0, 25.6, and 20.6). Therefore, in addition to one degree of unsaturation from the carbonyl group, three remaining ones could indicate the presence of a tricyclic system in **1**.

**Figure 2:**
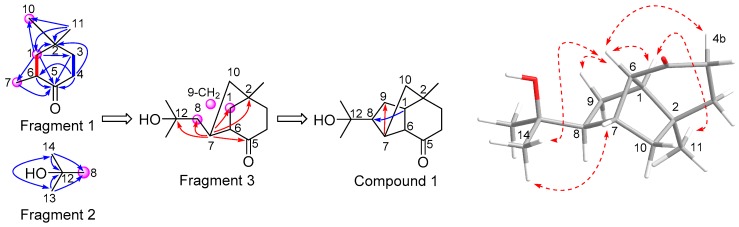
Key COSY (bond lines), HMBC (arrows), and NOE (dashed lines) correlations of **1** *^a^*. (*^a^* blue and red lines represented NMR signals determining CDCl_3_ and CD_3_OD, respectively).

Firstly, the obvious COSY correlations between H-3a and H_2_-4 could confirm a structural fragment of CH_2_(3)-CH_2_(4) (Figure 2 and Figure S6). Further, due to the partially overlapped ^1^H NMR signals of H-6 and H-3b, another CH(1)-CH(6) or CH_2_(3) could be deduced by the COSY cross-peak from H-1 to H-6 or 3b (Figure S6). In order to clearly distinguish H-6 and 3b in the NMR spectra, the novel **1** was further determined using CD_3_OD with a higher sensitivity of 600 MHz. As shown in Figure 2 and Figure S11, the fragment could be unambiguously confirmed as CH(1)-CH(6) or CH(1)-CH_2_(3). However, the NMR signals of H-8, H_2_-10 and 9 were still seriously overlapped using CDCl_3_ (Figures S2 and S6) or CD_3_OD (Figures S8 and S11), which were therefore difficult to clearly distinguish for the structure elucidation.

Secondly, the presence of a cyclohexanone residue (fragment 1) could be deduced by two groups of key HMBC correlations (Figure 2 and Figure S7), one including HMBC signals from H-1/6 and H_2_-3/4 to CO-5 (Figure 2 and Figure S7A), while another containing HMBC correlations from H-1, H_2_-4, H_3_-11 to C-2, from H_3_-11 to CH-1, from H-1 to CH_2_-3, and from H_2_-4 to CH-6 (Figure 2 and Figure S7B). The connection between the methylene residue (*δ*_C_ 35.9, CH_2_-10) and the quaternary carbon (C-2) could be confirmed by the HMBC cross-peaks from H-1 and H_3_-11 to CH_2_-10. Fragment 2 could also be deduced by the HMBC correlations shown in Figure 2 and Figure S7B.

Thirdly, as shown in Figure 2 and Figure S12, the obvious HMBC correlations from H-7 to CH-1 and CO-5 could indicate that the CH-7 group was connected to CH-6 of the cyclohexanone residue. The significant HMBC cross-peaks from H-7 to C-2, as well as from H-7 to CH-8 and C-12, could join fragments 1 and 2 to obtain fragment 3, through a CH(7)-CH_2_(10)-C2 bridge and a CH7-CH8 bond, respectively (Figure 2 and Figure S12B).

Finally, the last cyclopentane residue could be formed through the CH1-CH_2_(9)-CH8 linkage as evidenced by the key HMBC correlations from H-1 to CH-8 and from H-7 to CH_2_-9 (Figure 2 and Figure S12B). Therefore, based on the analyses of MS and NMR data, the novel **1** could be determined as a norsesquiterpene with tricyclic-6/5/5-[4.3.1.0^1,6^]decane skeleton.

H-7 of novel **1** was nearby to H-6, 8 and H_2_-10, respectively, but only showed a clear singlet peak in ^1^H NMR spectrum, no matter which deuterated reagents of CDCl_3_ (500 MHz, Figure S2), CD_3_OD (600 MHz, Figure S8) and DMSO-*d*_6_ (500 MHz, Figure S14) had been applied. Therefore, based on the Karplus-type equation showing the relationship between the coupling constant and dihedral angles [23,24], the singlet peak of H-7 should be related to the nearly perpendicular angles of nearby hydrogens of H-6, 8 and H_2_-10 (Figure 2). Similar results have been reported in many terpenoids, such as CH-8 (*δ*_H_ 2.15, dd, 4.8, 3.9) in sesquiterpene of penicibilaene A [25] and CH-7 (*δ*_H_ 3.81, d, 4.9) in diterpene of conidiogenone G [26].

As shown in Table 1 and Figure S8B, the large 5.6 Hz of H-6 was the vicinal coupling between H-6 (a bond) and H-1 (e bond), while the small 2.1 Hz of H-6 should be a long-range coupling between H-6 and H-4b, which usually appeared in bridged-ring or unsaturated compounds. The H-1 could be observed as a doublet peak with a coupling constant of 5.6 Hz, which was identical to that of H-6 (Figure S8B). This “d” peak of H-1 indicated that there was no vicinal coupling between H-1 and H_2_-9, probably due to their mutual nearly perpendicular angles. The lack of some COSY correlations of H-7 and H_2_-9 (Figures S6 and S11) should also be related to their perpendicular positions to nearby hydrogens [25].

The ^13^C NMR shift of CH-1 was assigned as unusually large, 66.1, which should be related to the anisotropic deshielding effects of nearby C-C bonds, while the shift of CH-6 (*δ*_C_ 37.1) to a higher field might be connected with the anisotropic shielding effect of the C=O bond, which also resulted in the higher-field-shifted CH_2_-4 (*δ*_C_ 20.1) compared with CH_2_-3 (*δ*_C_ 33.0). Similar results were usually found in bridgehead carbons of polycyclic terpenoids, such as CH-5 (*δ*_C_ 61.5) in penicibilaene A [25], as well as CH-6 (*δ*_C_ 66.0) and CH-15 (*δ*_C_ 70.1) in conidiogenone G [26].

**Figure 3:**
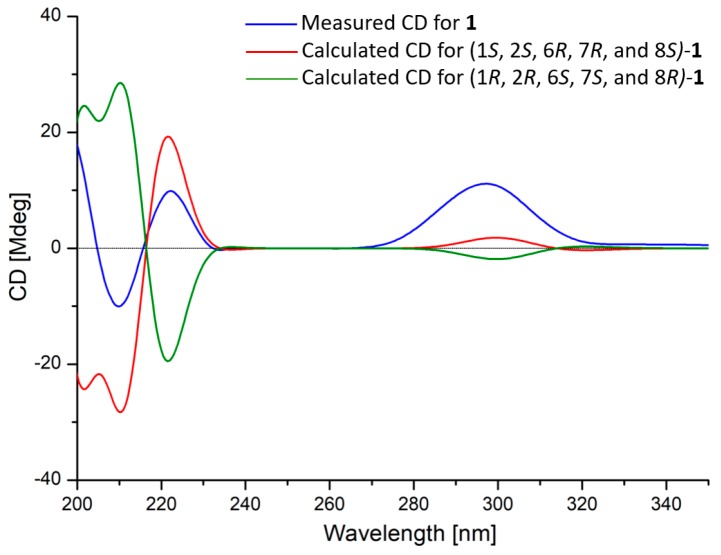
Comparisons of calculated ECD spectra for (1*S*, 2*S*, 6*R*, 7*R*, and 8*S*) and (1*R*, 2*R*, 6*S*, 7*S*, and 8*R*) with the experimental one of compound **1** in CH_3_OH.

The relative configuration of compound **1** was deduced through the NOESY experiment (Figure 2 and Figure S13). The consecutive NOE correlations from H_3_-11 to H-1, from H-1 to H-6, from H-6 to H-4b, H*_axial_*-9b and H_3_-14, as well as from H_3_-14 to H-7, suggested the co-face orientation of these protons. The absolute configuration of **1** was determined by the comparison of its experimental and calculated ECD (Electronic Circular Dichroism) spectra. As shown in Figure 3, the calculated ECD data of (1*S*, 2*S*, 6*R*, 7*R*, and 8*S*)-**1** showed positive cotton effects (CEs) near 220 and 295 nm, as well as negative CE around 210 nm, the same as the experimental ones, while the calculated ECD spectra of (1*R*, 2*R*, 6*S*, 7*S*, and 8*R*)-**1** exhibited opposite corresponding CEs.

The molecular formula of compound **1** only showed fourteen carbons, one less than the fifteen of the general sesquiterpenes, and therefore belongs to the rare norsesquiterpene. Compound **1** also possessed an unprecedented tricyclic-6/5/5-[4.3.1.0^1,6^]decane skeleton, especially with a C2-C10-C7 bridge across two carbon rings. The ring system of **1** was similar to that of the pupukeanan skeleton (tricyclic-6/5/6-[4.3.1.0^3,7^]decane) with its carbon bridge located in the contiguous carbon of the cyclohexanone residue in 1, which also suggested that they should be biosynthesized with similar pathways [27,28,29,30]. To our best knowledge, the carbon skeleton of novel **1** was first reported in the norsesquiterpene series and could be named as norpupukeanane skeleton. Therefore, the novel **1** could be named as norpupukeanane A, which was also first reported from marine-derived fungus. Its proposed biosynthesis via mevalonic acid pathway might be similar to that of previous norsesquiterpenes [31,32].

The isolation of antagonistic fractions from the culture extract also resulted in other known metabolites, including sesquiterpenes (**2**–**6**) and cyclodepsipeptides (**7**–**9**). Their structures were determined by detailed analyses of their spectroscopic data and comparisons with previously published reports as follows: trichothecinol A (**2**) [33], 8-deoxy-trichothecin (**3**) [34], trichothecinol B (**4**) [33], 10-cycloneren-3,5,7-triol (**5**) [17], 10(*E*)-cyclonerotriol (**6**) [35], homodestcardin (**7**) [11], trichomide B (**8**) [11], and homodestruxin B (**9**) [11]. Compounds **2**–**4** and **5** and **6** belong to sesquiterpenes of the trichothecene and cyclonerodiol series, respectively, while **7**–**9** are attributed to cyclohexadepsipeptides of the trichomide series. All the known metabolites (**2**–**9**) were first reported to be isolated from the species of *T. longibrachiatum*.

### 2.2. Antagonistic Evaluation

The isolated metabolites of sesquiterpenes **1**–**6** and cyclodepsipeptides **7**–**9** were evaluated for their antagonistic potential (Table 2), including antifungal activities against three groups of representative soil-borne phytopathogenic fungi—*Colletotrichum lagrnarium*, *Colletotrichum fragariae*, carbendazim-resistant strains of *Botrytis cinerea* from grape (PTQ1) and strawberry (CMQ1), *Fusarium oxysporum* f. sp. *cucumerinum*, and *Fusarium oxysporum* f. sp. *Lycopersici*—as well as nematicidal effects against the southern root-knot nematode (*M. incognita*).

The novel norsesquiterpene **1** showed significant antifungal activities against two *Colletotrichum* species and two carbendazim-resistant strains of *B. cinerea* with MIC values ranging from 8 to 64 μg/mL. These results are better than those from the commonly used carbendazim, a benzimidazole fungicide which binds to the *β*-tubulin proteins and then inhibits cell division [36]. Therefore, although there are almost no reports concerning antifungal mechanisms of norsesquiterpenes, the novel **1** could show multiple-target potential.

The known trichothecene sesquiterpenes **2** and **3** exhibited broad-spectrum antifungal activities against all tested soil-borne phytopathogenic fungi, while the trichothecene congener **4** only showed far weaker effects compared to those of **2** and **3**, suggesting that the 4-*OH* substituted in the cyclohexane ring of **4** might negatively modulate its antifungal activity. Trichothecenes are known as mycotoxins, which show antifungal, phytotoxic and cytotoxic activities [37,38,39]. Antifungal SAR research of trichothecene congeners showed that the 12-epoxide was essential to its activity [37], while the substituted groups in C-4 and C-8 could also modulate the effect [38], which was identical to the SAR of isolated **2**–**4**. *Trichoderma* trichothecenes have been reported to be able to induce the expression of plant defense-related genes [39].

Trichomide cyclodepsipeptides **7**–**9** exhibited moderate nematicidal activities against *M. incognita*. Cyclonerodiol sesquiterpenes **5** and **6** could also partially kill the second-stage juveniles (J2s) of *M. incognita* at concentration of 200 μg/mL. Although the metabolites **5**–**9** showed weaker nematicidal potential compared to the positive control of abamectin, they could also exert synergistic effects with the antifungal **1**–**4** for the control of soil-borne diseases. The antifungal and nematicidal potential of known metabolites (**2**–**9**) was also first reported.

## 3. Experimental Section

### 3.1. General Procedures

One- and two-dimensional NMR spectra were recorded at 500 and 125 MHz for ^1^H and ^13^C, respectively, using a Bruker Avance III spectrometer (Bruker Biospin Group, Karlsruhe, Germany) with TMS as internal standard. HRESIMS data were determined on a mass spectrometer, the Thermo Scientific Orbitrap Fusion Lumos Tribrid (Thermo Scientific, Waltham, MA, USA). Optical rotation was obtained using a Jasco P-1020 digital polarimeter (Jasco Corporation, Tokyo, Japan). CD spectrum was acquired on a Chirascan spectropolarimeter (Applied Photophysics Ltd., Surrey, UK). Column chromatography (CC) was performed with Si gel (200–300 mesh; Qingdao Haiyang Chemical Co., Qingdao, China), Lobar LiChroprep RP-18 (40–63 μm; Merck, Kenilworth, NJ, USA), and Sephadex LH–20 (18–110 μm; Merck). Semi-preparative HPLC (semi-pHPLC) was performed using a Dionex HPLC system equipped with a P680 pump (flow rate: 3 mL/min), an ASI-100 automated sample injector, and a UVD340U multiple wavelength detector (Detection wavelength: 220 nm) controlled using Chromeleon software, version 6.80 (Dionex Corporation, Sunnyvale, CA, USA).

### 3.2. Fungal Material

The fungal strain *T. longibrachiatum* was isolated from the root of *Suaeda glauca*, a highly halophile plant collected from the intertidal zone of Jiaozhou Bay, Qingdao, China in October 2015. The fungus was identified on the basis of morphological characteristics and molecular analyses of ITS (internal transcribed spacer)-5.8S Rdna region sequence [20]. The strain was preserved in the Natural Products Laboratory, College of Chemistry and Pharmacy, Qingdao Agricultural University.

### 3.3. Fermentation, Extraction and Isolation

Fresh mycelia of the fungus were statically fermented at 28 °C for 30 days on liquid Potato Dextrose Broth (PDB) media. The liquid culture was conducted in 50 × 1 L conical flasks containing 300 mL of PDB medium (2% glucose and 20% potato juice in natural seawater).

The PDB culture was exhaustively extracted using EtOAc to obtain a crude extract, which was further fractionated via silica gel vacuum liquid chromatography (VLC) with petroleum ether/acetone (20:1, 10:1, 5:1, and 1:1) and chloroform/MeOH gradients (20:1, 10:1, 5:1, and 1:1) to yield eight fractions (Frs. 1–8). The potential antagonistic Fr. 4 was purified via CC over RP-C18, eluting with a MeOH-H_2_O gradient (from 1:9 to 1:0) to obtain five subfractions (Fr. 3-1 to 3-5). Fr. 3-3 was first separated using Sephadex LH-20 (MeOH), and then isolated via CC over silica gel with a chloroform/MeOH gradient (from 40:1 to 10:1) to yield compounds **5** (5.6 mg) and **6** (11.3 mg). Fr. 3-4 was purified via CC over Sephadex LH-20 (MeOH) and further separated using semi-pHPLC (45% MeCN-H_2_O) to obtain compounds **2** (6.5 mg, t*_R_* 13.4 min), **3** (9.6 mg, t*_R_* 18.1 min) and **4** (7.7 mg, t*_R_* 15.8 min). Fr. 3-5 was isolated using Sephadex LH-20 (Acetone) to yield compound **1** (3.1 mg) and a subfraction, which was further purified via semi-pHPLC (70% MeOH-H_2_O) to obtain compounds **7** (8.5 mg, t*_R_* 16.7 min), **8** (3.4 mg, t*_R_* 18.1 min) and **9** (10.2 mg, t*_R_* 21.4 min).

*Trichodermene A* (**1**): Colorless oil. [*α*${]}_{D}^{24}$= +7.4, *c* 1.20, CH_3_OH; UV (CH_3_OH) *λ*_max_ (log *ε*) 205 (2.28), 284 (1.05) nm; IR (CH_3_OH) *ν*_max_ 3431, 3257, 2945, 2832, 1652, 1043 cm^−1^; ECD (CH_3_OH) *λ*_max_ ([θ]) 210 (–10.05), 222 (+9.90), 297 (+11.13) nm; ^1^H and ^13^C NMR data, see Table 1; HRESIMS *m/z* 245.1489 [M + Na]^+^ (calcd for NaC_14_H_22_O_6_, 245.1484).

### 3.4. Antagonistic Evaluation

In order to evaluate the antagonistic potential of *T. longibrachiatum* metabolites against soil-borne pathogens, its antifungal activities were tested against three groups of representative soil-borne phytopathogens, including *Colletotrichum* spp. (*C. fragariae* and *C. lagenarium*), carbendazim-resistant strains of *B. cinerea* from grape (PTQ1) and strawberry (CMQ1), and *Fusarium oxysporum*, using a broth microdilution method in 96-well plates [21,40].

Another synergetic soil-borne pathogen, the southern root-knot nematode (*M. incognita*), was also selected for nematicidal bioassay in 24-well plates. Briefly, J2s of *M. incognita* were collected to prepare the nematode suspension based on the protocol reported previously [21]. The isolated metabolites (**1**–**9**) were dissolved and diluted in DMSO to obtain sample solvents with a series of different concentrations. The sample solvents (5 μL) were added to each well containing the nematode suspension (495 μL) with about 60 J2s, while the same amount of DMSO (5 μL) was added for the negative control. The plates were maintained at 25 °C for 48 h and then observed using a stereomicroscope to evaluate the nematode mortalities. Nematodes were defined to be dead if their bodies became straight and did not react to mechanical touches. The experiment was repeated three times under the same conditions.

## 4. Conclusions

The investigation of antagonistic metabolites from marine-derived fungus *T. longibrachiatum* obtained two structural series of sesquiterpenes **1**–**6** and cyclodepsipeptides **7**–**9**. Notably, the novel **1** was a rare norsesquiterpene possessing an unprecedented tricyclic-6/5/5-[4.3.1.0^1,6^]decane skeleton. Its absolute configuration was determined by the comparison of experimental and calculated ECD spectra. The novel **1** and known **2** and **3** showed significant antifungal activities against two *Colletotrichum* species and two carbendazim-resistant strains of *B. cinerea* with MIC values ranging from 8 to 64 μg/mL, even better than those of the commonly used fungicide carbendazim. Cyclodepsipeptides **7**–**9** showed moderate nematicidal activities against the southern root-knot nematode (*M. incognita*). The antifungal activities of **1**–**4** and nematicidal effects of **5**–**9** were reported for the first time and further revealed the synergistically antagonistic potential of marine-derived *T. longibrachiatum* against soil-borne pathogens.

## Figures and Tables

**Table 1:** ^1^H and ^13^C NMR data of compound **1** (*δ*: ppm).

	Compound 1 *^a^*		Compound 1 *^b^*	
No.	*δ*_C_ (type)	*δ*_H_ (mult., *J* in Hz)	*δ*_C_ (type)	*δ*_H_ (mult., *J* in Hz)
1	66.1, CH	2.24, br. s	68.4, CH	2.15, d (5.6)
2	43.4, C		45.6, C	
3	33.0, CH_2_	2.56, m; 2.37, ov	34.8, CH_2_	2.61, ddd (18.2, 11.4, 6.6) 2.34, ddd (18.2, 9.5, 1.9)
4a 4b	20.1, CH_2_	2.13, m; 2.02, m	21.73, CH_2_	2.19, ov; 2.03, dddd (13.8, 9.5, 6.6, 2.1)
5	214.7, CO		219.0, CO	
6	37.1, CH	2.39, ov	39.2, CH	2.40, dt (5.6, 2.1)
7	37.5, CH	2.40, s	39.6, CH	2.46, s
8	48.0, CH	1.83, ov	50.4, CH	1.80, ov
9	20.3, CH_2_	1.73, ov	22.2, CH_2_	1.77, ov
10	35.9, CH_2_	1.78, ov	38.0, CH_2_	1.83, ov; 1.72, ov
11	20.6, CH_3_	0.87, s	21.71, CH_3_	0.84, s
12	73.6, C		75.0, C	
13	28.0, CH_3_	1.23, s	28.5, CH_3_	1.18, s
14	25.6, CH_3_	1.19, s	25.7, CH_3_	1.16, s

*^a,b^* Compound **1** was determined using CDCl_3_ *^a^* (^1^H of 500 MHz and ^13^C of 125 MHz) and CD_3_OD *^b^* (^1^H of 600 MHz and ^13^C of 150 MHz), respectively; ov: overlapped ^1^H NMR signals.

**Table 2:** Antifungal (MIC: μg/mL) and nematicidal (IC_50_: μg/mL) activities of the isolated metabolites **1**–**9**.

Compounds	*C. lagrnarium*	*C. fragariae*	PTQ1	CMQ1	*F. oxysporum* f. sp. *cucumerinum*	*F. oxysporum* f. sp. *lycopersici*	*M. incognita*
**1**	8	16	64	32	128	256	—
**2**	16	64	32	32	64	32	—
**3**	16	64	64	32	32	32	—
**4**	64	128	256	128	128	256	—
**5**	—	—	—	—	—	—	38.2% *^c^*
**6**	—	—	—	—	—	—	42.7% *^c^*
**7**	—	—	256	—	—	—	149.2
**8**	—	—	—	—	—	—	140.6
**9**	—	—	—	256	—	—	198.7
Carbendazim *^a^*	32	16	256	256	4	8	
Abamectin *^b^*							24.9

“—”: no activity. *^a,b^* carbendazim and abamectin for antifungal and nematicidal bioassays, respectively. *^c^* J2s lethal rate of *M. incognita* at concentration of 200 μg/mL.

## Supplementary Materials

The following are available online at https://www.mdpi.com/1660-3397/18/3/165/s1, Figure S1: HRESIMS spectrum of compound **1**; Figures S2–S14: annotated 1D NMR and selected 2D NMR spectra of compound **1**; Experimental Section: The method of calculated ECD spectrum of compound **1**.

## References

[1] Ralph D., Jan A.L.V.K., Zacharias A.P., Ki E.H., Antonio D.P., Pietro D.S., Jason J.R., Marty D., Regine K., Jeff E. (2012). The top 10 fungal pathogens in molecular plant pathology. Mol. Plant Pathol..

[2] Liu T.T., Wu H.B., Jiang H.Y., Zhang L., Zhang Y.N., Mao L.G. (2019). Thiophenes from *Echinops grijsii* as a preliminary approach to control disease complex of root-knot nematodes and soil-borne fungi: Isolation, activities, and structure−nonphototoxic activity relationship analysis. J. Agric. Food Chem..

[3] Muhammad Z.K., Tariq M., Muhammad A.H. (2017). Effects of southern root knot nematode population densities and plant age on growth and yield parameters of cucumber. Crop. Prot..

[4] Huang S.Z., Huang H.N., Ma Q.Y., Mo M.H., Zhu M.L., Dai H.F., Ji Y.P., Wang Q.H., Zhao Y.X. (2015). The phytochemicals with antagonistic activities toward pathogens of a disease complex caused by *Meloidogyne incogni* and *Ralstonia solanacearumta*. J. Pure Appl. Microbiol..

[5] Van der Putten W.H., Dijk C.V., Peters B.A.M. (1993). Plant-specific soil-borne diseases contribute to succession in foredune vegetation. Nature.

[6] Russell P.E. (2006). The development of commercial disease control. Plant Pathol..

[7] Charles L.C., Franck E.D., Stephen O.D. (2012). Natural products as sources for new pesticides. J. Nat. Prod..

[8] Vincenzo M., Aurélie S., Enrico B., Catia P., Cindy C., Patricia T.A., Christophe C., Laura M., Florence F. (2018). Grapevine trunk diseases: A review of fifteen years of trials for their control with chemicals and biocontrol agents. Plant Dis..

[9] Rosa H., Ada V., Ilan C., Enrique M. (2012). Plant-beneficial effects of *Trichoderma* and of its genes. Microbiology.

[10] Morton C.O., Penny R.H., Brian R.K. (2004). Infection of plant-parasitic nematodes by nematophagous fungi—A review of the application of molecular biology to understand infection processes and to improve biological control. Nematology.

[11] Nashwa M.A.S., Amal M.I.E., Ahmed S. (2019). Effect of *Trichoderma* spp. On fusarium wilt disease of tomato. Mol. Biol. Rep..

[12] Kamal A.M.A., Sobhy I.I.A., Ismail R.A. (2014). Isolation of Trichoderma and evaluation of their antagonistic potential against *Alternaria porri*. J. Phytopathol..

[13] AL-Shammari T.A., Bahkali A.H., Elgorban A.M., El-Kahky M.T., Al-Sum B.A. (2013). The use of *Trichoderma longibrachiatum* and *Mortierella alpina* against root-knot nematode, *Meloidogyne javanica* on tomato. J. Pure Appl. Microbiol..

[14] Cesar G.O., Víctor G.P., Stefania L., Matteo L. (2014). Enzyme activity of extracellular protein induced in *Trichoderma asperellum* and *T. longibrachiatum* by substrates based on *Agaricus bisporus* and *Phymatotrichopsis omnivora*. Fungal Biol..

[15] Song Y.P., Miao F.P., Liu X.H., Ji N.Y. (2019). Responses of marine-derived *Trichoderma* fungi to seawater and their potential antagonistic behavior. J. Oceanol. Limnol..

[16] Song Y.P., Shi Z.Z., Miao F.P., Fang S.T., Yin X.L., Ji N.Y. (2018). Tricholumin A, a highly transformed ergosterol derivative from the alga-endophytic fungus *Trichoderma asperellum*. Org. Lett..

[17] Song Y.P., Fang S.T., Miao F.P., Yin X.L., Ji N.Y. (2018). Diterpenes and sesquiterpenes from the marine algicolous fungus *Trichoderma harzianum* X-5. J. Nat. Prod..

[18] Shi Z.Z., Fang S.T., Miao F.P., Yin X.L., Ji N.Y. (2018). Trichocarotins A–H and trichocadinin A, nine sesquiterpenes from the marine-alga-epiphytic fungus *Trichoderma virens*. Bioorg. Chem..

[19] Liang X.R., Miao F.P., Song Y.P., Liu X.H., Ji N.Y. (2016). Citrinovirin with a new norditerpene skeleton from the marine algicolous fungus *Trichoderma citrinoviride*. Bioorg. Med. Chem. Lett..

[20] Du F.Y., Li X., Li X.M., Zhu L.W., Wang B.G. (2017). Indolediketopiperazine alkaloids from *Eurotium cristatum* EN-220, an endophytic fungus isolated from the marine alga *Sargassum thunbergii*. Mar. Drugs.

[21] Zhou Y.M., Ju G.L., Xiao L., Zhang X.F., Du F.Y. (2018). Cyclodepsipeptides and sesquiterpenes from marine-derived fungus *Trichothecium roseum* and their biological functions. Mar. Drugs.

[22] Xiao L., Zhou Y.M., Zhang X.F., Du F.Y. (2018). Antifungal activity against apple fruit pathogens of *Notopterygium incisum* extract and bioactive secondary metabolites. Pestic. Biochem. Phys..

[23] Yuan X.R., Duan E.H., Zhang Y., Hu S., Liu Y.H. (2010). Determining the configuration of trans-4-propyl-cyclohexylcarboxylic acid by NMR method. Mol. Cryst. Liq. Cryst..

[24] Abraham R.J., Leonard P., Tormena C.F. (2012). 1H NMR spectra. Part 28: Proton chemical shifts and couplings in three-membered rings. A ring current model for cyclopropane and a novel dihedral angle dependence for ^3^*J*_HH_ couplings involving the epoxy proton. Magn. Reson. Chem..

[25] Meng L.H., Li X.M., Liu Y., Wang B.G. (2014). Penicibilaenes A and B, sesquiterpenes with a tricyclo[6.3.1.0^1,5^]dodecane skeleton from the marine isolate of *Penicillium bilaiae* MA-267. Org. Lett..

[26] Du L., Li D.H., Zhu T.J., Cai S.X., Wang F.P., Xiao X., Gu Q.Q. (2009). New alkaloids and diterpenes from a deep ocean sediment derived fungus *Penicillium* sp.. Tetrahedron.

[27] Chang N.C., Chang C.K. (1996). Total synthesis of (±)-2-pupukeanone. J. Org. Chem..

[28] Liu L., Liu S.C., Jiang L.H., Chen C.L., Guo L.D., Che Y.S. (2008). Chloropupukeananin, the first chlorinated pupukeanane derivative, and its precursors from *Pestalotiopsis fici*. Org. Lett..

[29] Liu L., Niu S.B., Lu X.H., Chen X.L., Zhang H., Guo L.D., Che Y.S. (2010). Unique metabolites of *Pestalotiopsis fici* suggest a biosynthetic hypothesis involving a Diels–Alder reaction and then mechanistic diversification. Chem. Commun..

[30] Liu L., Bruhn T., Guo L.D., Gçtz D.C.G., Brun R., Stich A., Che Y.S., Bringmann G. (2011). Chloropupukeanolides C–E: Cytotoxic pupukeanane chlorides with a spiroketal skeleton from *Pestalotiopsis fici*. Chem. Eur. J..

[31] Shen T., Qian H., He Y.L., Li L.M., Wang Y.D. (2018). Longifodiol, a novel rearranged triquinane norsesquiterpene from the root of *Leontopodium longifolium*. Chem. Lett..

[32] Li B., Li L., Peng Z.G., Liu D., Si L.L., Wang J., Yuan B.C., Huang J., Proksch P., Lin W.H. (2019). Harzianoic acids A and B, new natural scaffolds with inhibitory effects against hepatitis C virus. Bioorg. Med. Chem..

[33] Akira I., Kazuhide K., Hiroki K., Kiyoshi T., Harukuni T., Hoyoku N. (1996). Trichothecinols A, B and C, potent anti-tumor promoting sesquiterpenoids from the fungus *Trichothecium roseum*. Tetrahedron Lett..

[34] Kenji T., Ronald D.P., Reiko Y., Masatoshi M., Masaru M., Shoichi K., Manfred G., Gen O. (2001). 8-deoxy-trichothecin production by *Spicellum roseum* isolated from a cultivated mushroom in Japan. Mycotoxins.

[35] Pang X.Y., Lin X.P., Tian Y.Q., Liang R., Wang J.F., Yang B., Zhou X.F., Kumaravel K., Luo X.W., Tu Z.C. (2018). Three new polyketides from the marine sponge derived fungus *Trichoderma* sp. SCSIO41004. Nat. Prod. Res..

[36] Yin W.X., Adnan M., Shang Y., Lin Y., Luo C.X. (2018). Sensitivity of *Botrytis cinerea* from nectarine/cherry in China to six fungicides and characterization of resistant isolates. Plant Dis..

[37] Matsumoto M., Nishiyama M., Maeda H., Tonouchi A., Konno K., Hashimoto M. (2019). Structure-activity relationships of trichothecenes against COLO201 cells and *Cochliobolus miyabeanus*: The role of 12-epoxide and macrocyclic moieties. Bioorg. Med. Chem. Lett..

[38] Cheng J.L., Zheng M., Yao T.T., Li X.L., Zhao J.H., Xia M., Zhu G.N. (2015). Synthesis, antifungal activity, and QSAR study of novel trichodermin derivatives. J. Asian Nat. Prod. Res..

[39] Malmierca M.G., Cardoza R.E., Alexander N.J., McCormick S.P., Hermosa R., Monte E., Gutiérrez S. (2012). Involvement of *Trichoderma* trichothecenes in the biocontrol activity and induction of plant defense-related genes. Appl. Environ. Microbiol..

[40] Zhang P., Yuan X.L., Du Y.M., Zhang H.B., Shen G.M., Zhang Z.F., Liang Y.J., Zhao D.L., Xu K. (2019). Angularly prenylated indole alkaloids with antimicrobial and insecticidal activities from an endophytic fungus *Fusarium sambucinum* TE-6L. J. Agric. Food Chem..

