# Supplementary material for: Sesquiterpenes and Cyclodepsipeptides from Marine-Derived Fungus Trichoderma longibrachiatum and Their Antagonistic Activities against Soil-Borne Pathogens
Source: Mar Drugs. 2020 Mar 16;18(3):165. doi: 10.3390/md18030165 (PMC7142749; doi:10.3390/md18030165)
Supplement: Supplementary file 1 [file marinedrugs-18-00165-s001.pdf]

Sesquiterpenes and Cyclodepsipeptides from Marine-Derived Fungus *Trichoderma Longibrachiatum* and Their Antagonistic Activities against Soil-borne Pathogens

Feng-Yu Du [1,2,†], Guang-Lin Ju [1,†], Lin Xiao [1], Yuan-Ming Zhou [3], and Xia Wu [4,*]

1 College of Chemistry and Pharmacy, Qingdao Agricultural University, Qingdao 266109, China; fooddfy@126.com (F.Y.); jgl2018666@163.com (G.J.); xiaolin_qd@163.com (L.X.)
2 Shandong Key Laboratory of Applied Mycology, Qingdao Agricultural University, Qingdao 266109, China
3 Analytical and Testing Center, Qingdao Agricultural University, Qingdao 266109, China; zym7410@163.com (Y.Z.)
4 Key Lab of Integrated Crop Pest Management of Shandong Province, College of Plant Health and Medicine, Qingdao Agricultural University, Qingdao 266109, China; wuxia3897@163.com (X.W.)
* Correspondence: wuxia3897@163.com (X.W.)
† These authors contributed equally to this work.

Contents

Figure S1. HRESIMS spectrum of compound **1**. S2
Figure S2. ^{1}H NMR (500 MHz, $CDCl_3$) spectrum of compound **1**. S3
Figure S3. ^{13}C NMR spectrum of compound **1**. S3
Figure S4. DEPT-135 spectrum of compound **1**. S4
Figure S5. HSQC spectrum of compound **1**. S4
Figure S6. ^{1}H-^{1}H COSY spectrum of compound **1**. S5
Figure S7. HMBC spectrum of compound **1**. S6
Figure S8. ^{1}H NMR (600 MHz, CD_3OD) spectrum of compound **1**. S7
Figure S9. ^{13}C NMR spectrum of compound **1**. S8
Figure S10. HSQC spectrum of compound **1**. S8
Figure S11. ^{1}H-^{1}H COSY spectrum of compound **1**. S9
Figure S12. HMBC spectrum of compound **1**. S10
Figure S13. NOESY spectrum of compound **1**. S11
Figure S14. ^{1}H NMR (500 MHz, DMSO-d_6) spectrum of compound **1**. S11
Experimental Section: Method of calculated ECD spectrum of compound **1**. S12

Figure S1. HRESIMS spectrum of compound **1**.

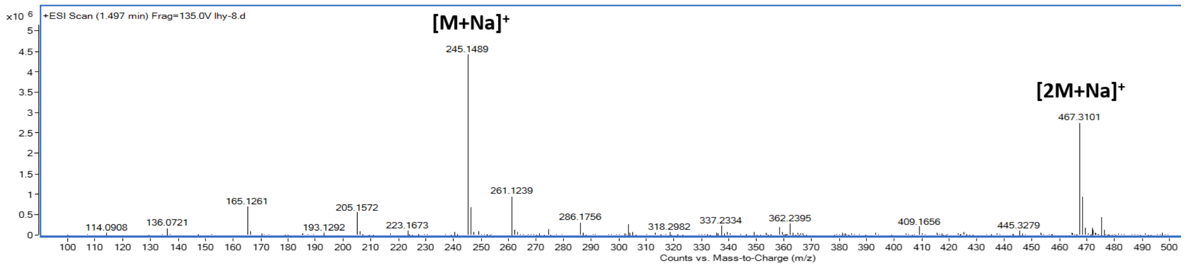

Figure S2. ^{1}H NMR (500 MHz, $CDCl_3$) spectrum of compound **1**.

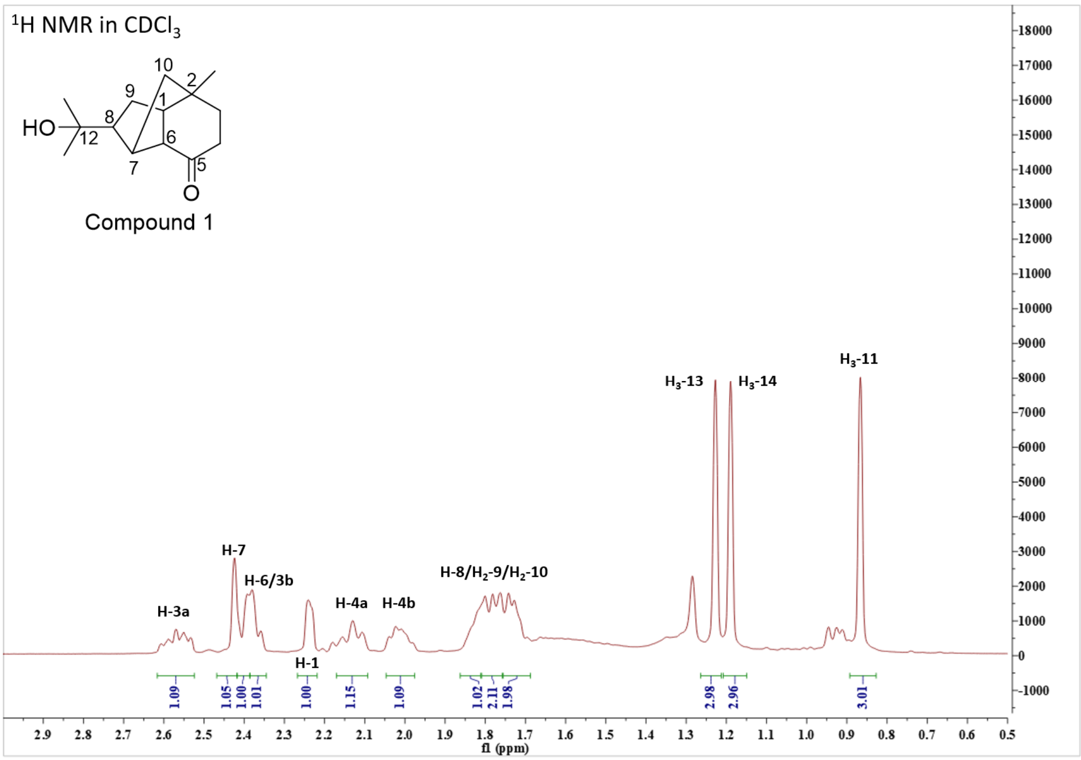

Figure S3. ^{13}C NMR spectrum of compound **1**.

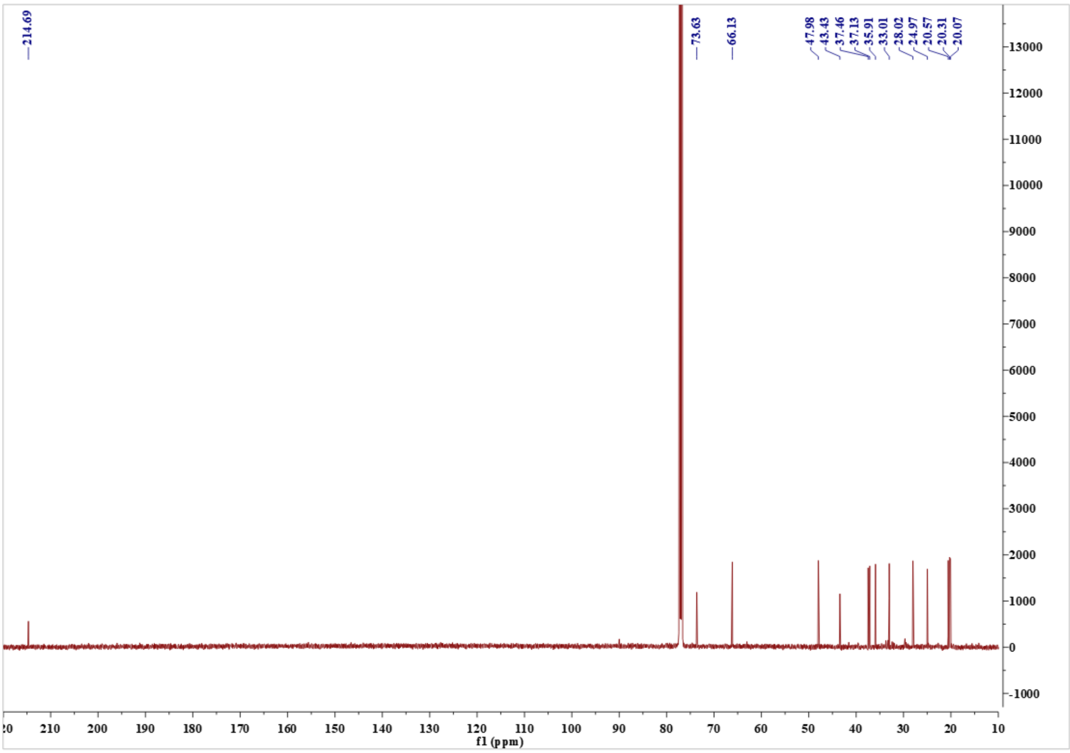

Figure S4. DEPT-135 spectrum of compound **1**.

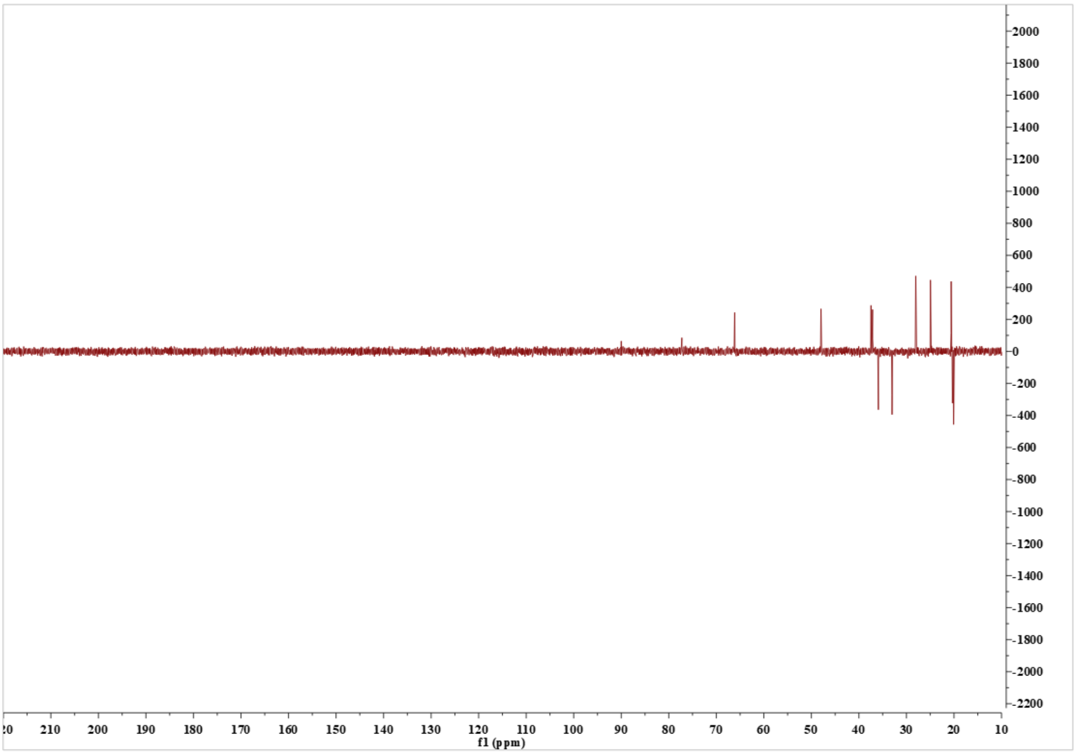

Figure S5. HSQC spectrum of compound **1**.

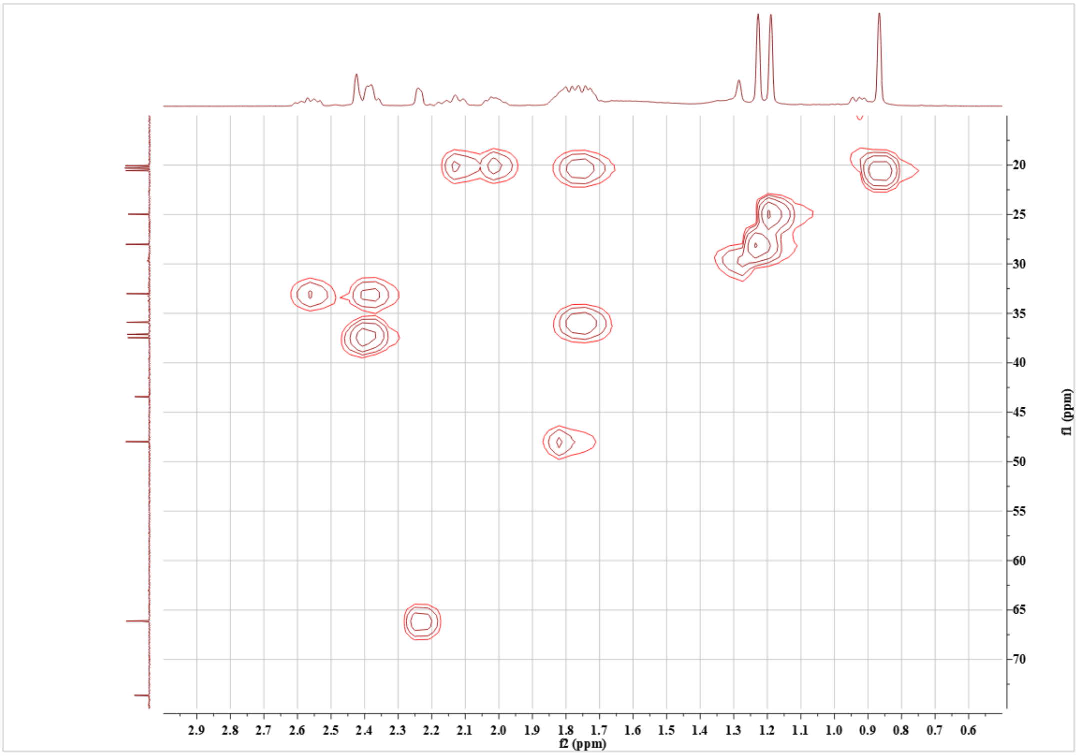

Figure S6. ^{1}H-^{1}H COSY spectrum of compound **1**.

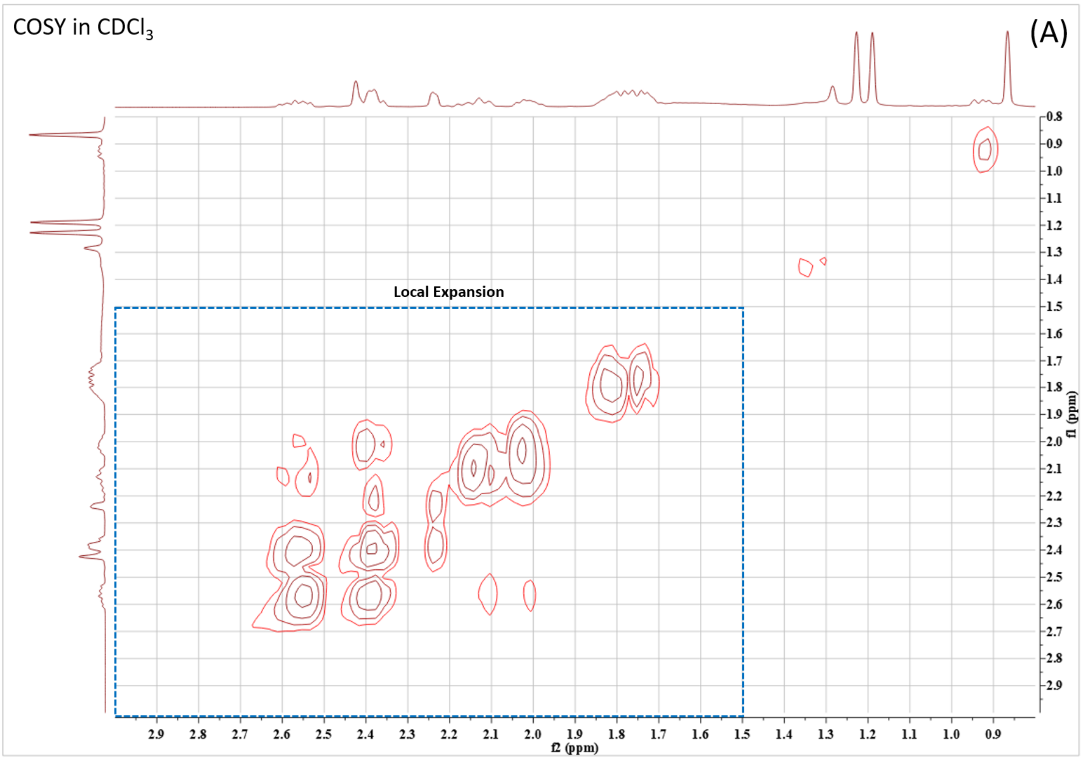

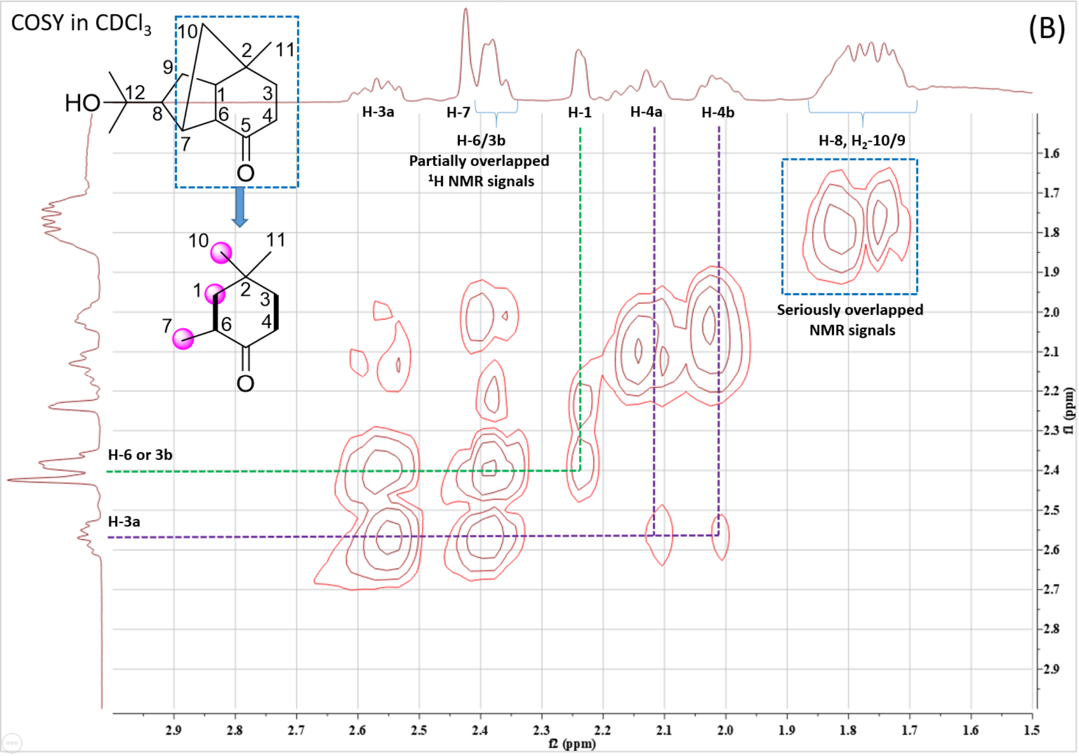

Figure S7. HMBC spectrum of compound **1**.

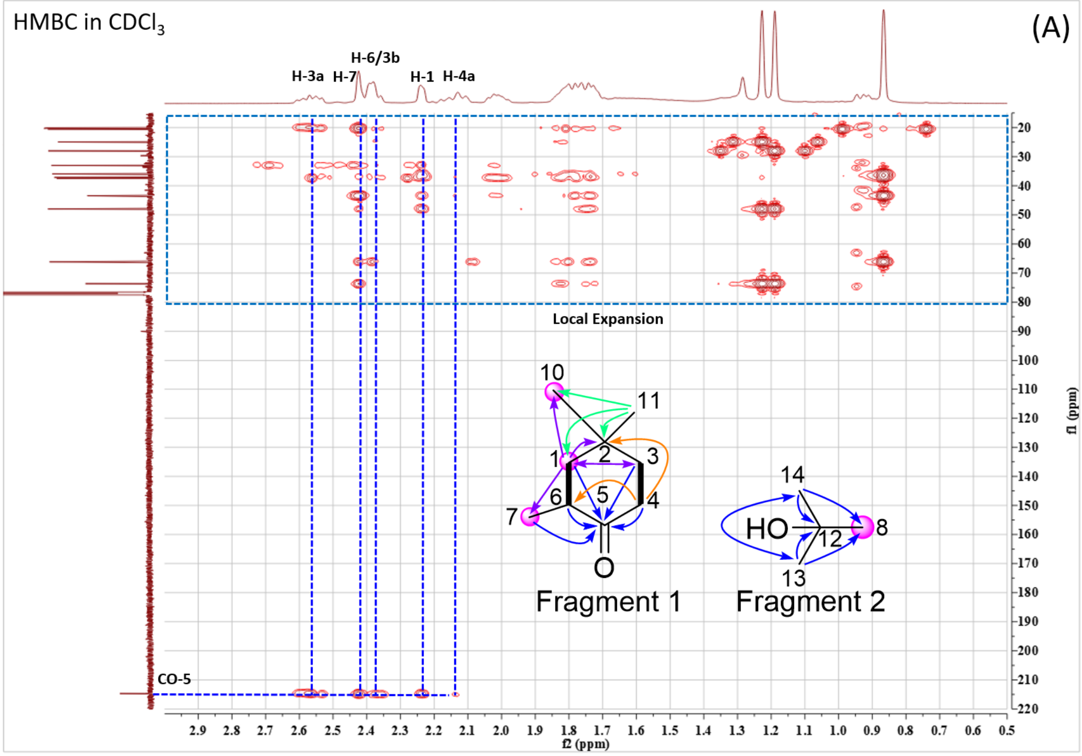

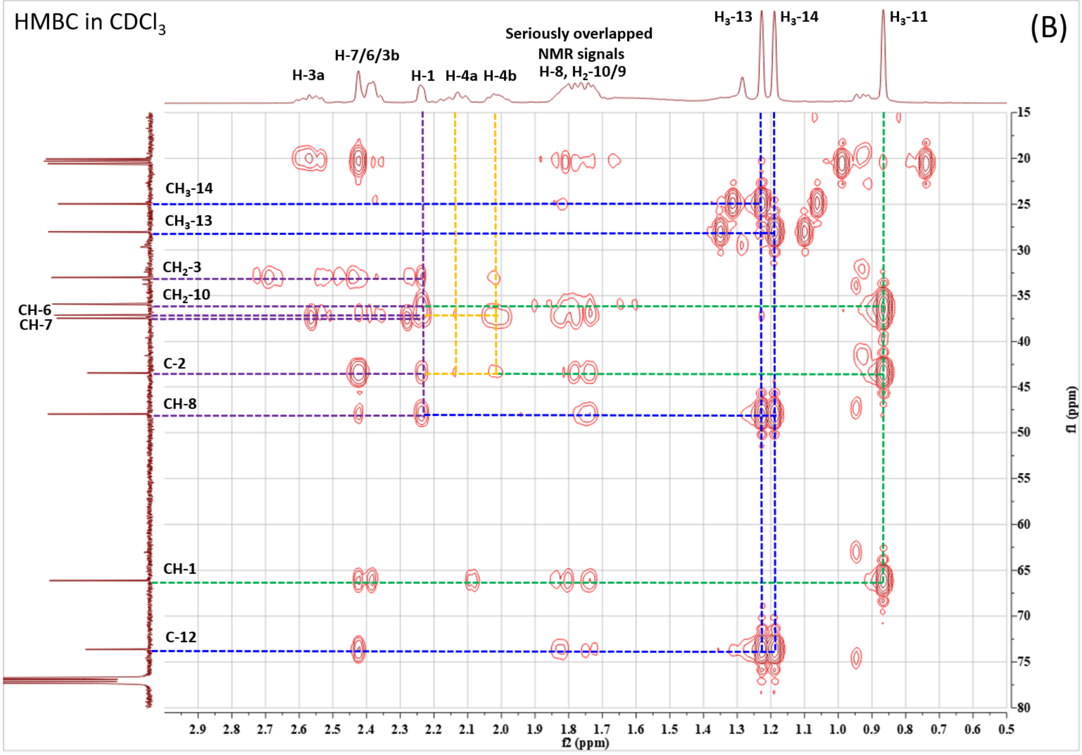

Figure S8. ^1H NMR (600 MHz, CD_3OD) spectrum of compound **1**.

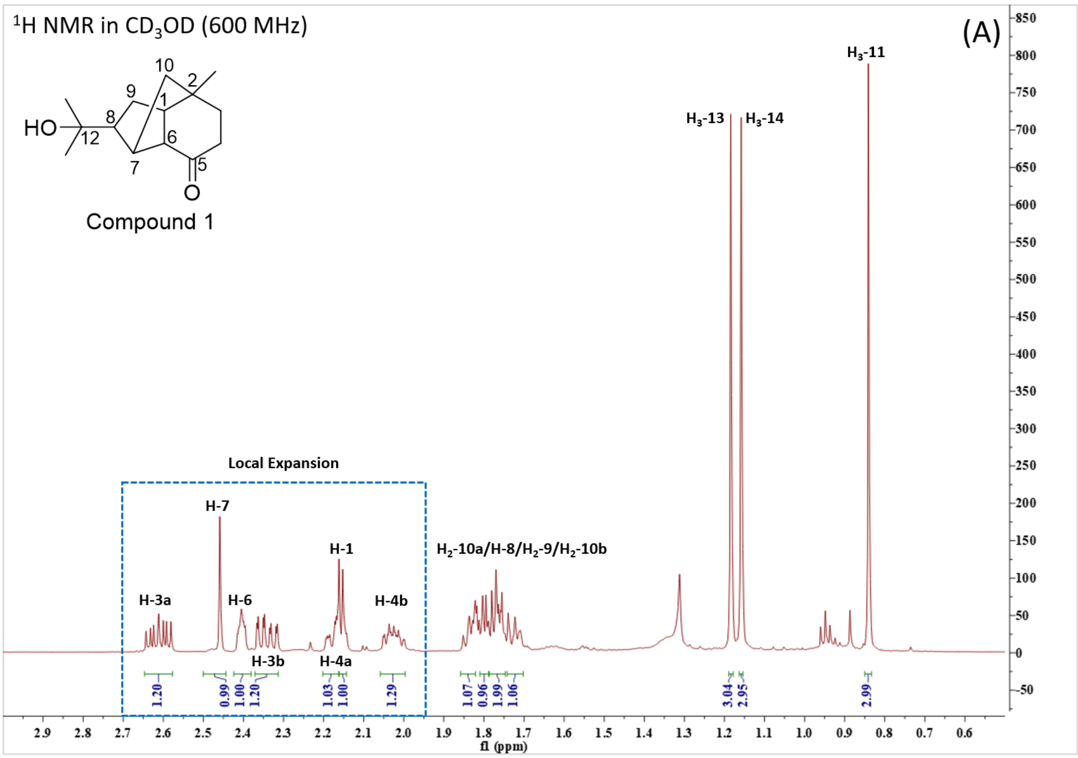

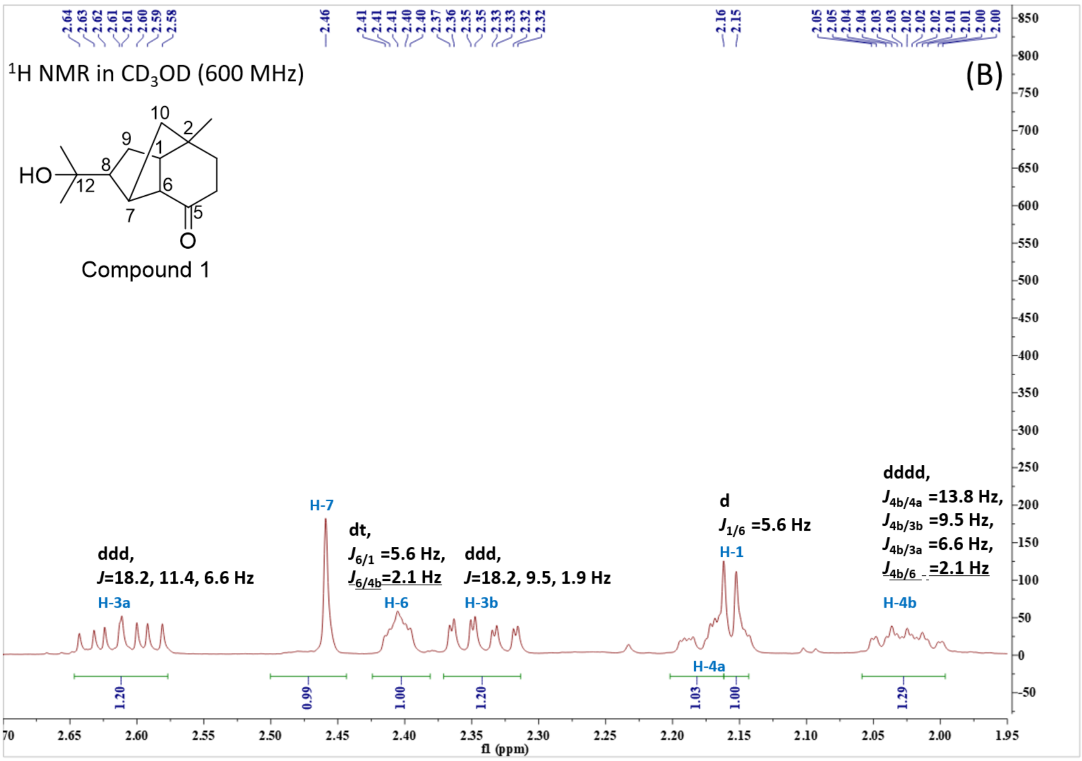

Figure S9. ^{13}C NMR spectrum of compound **1**.

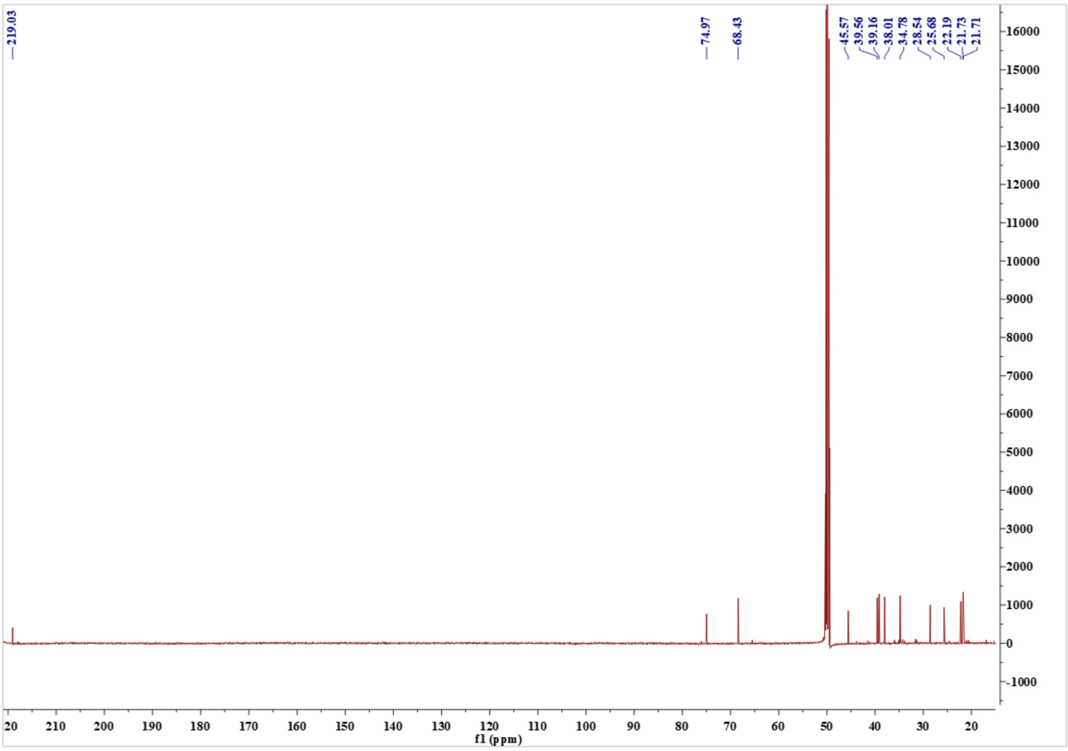

Figure S10. HSQC spectrum of compound **1**.

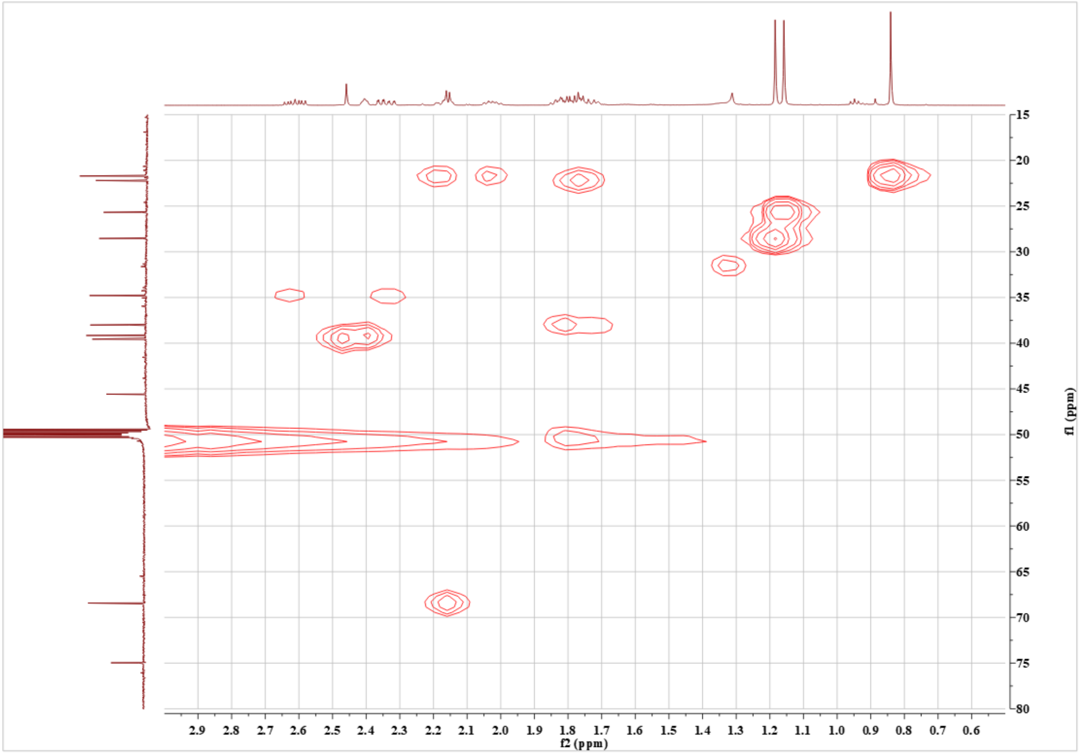

Figure S11. ^{1}H-^{1}H COSY spectrum of compound **1**.

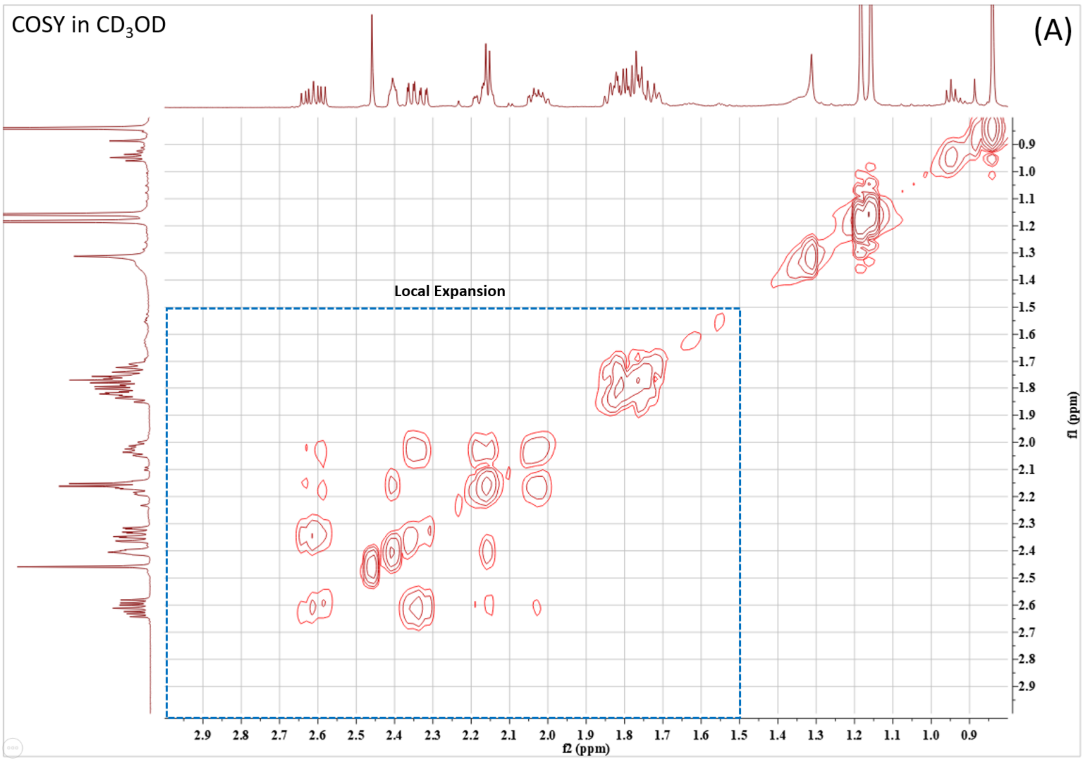

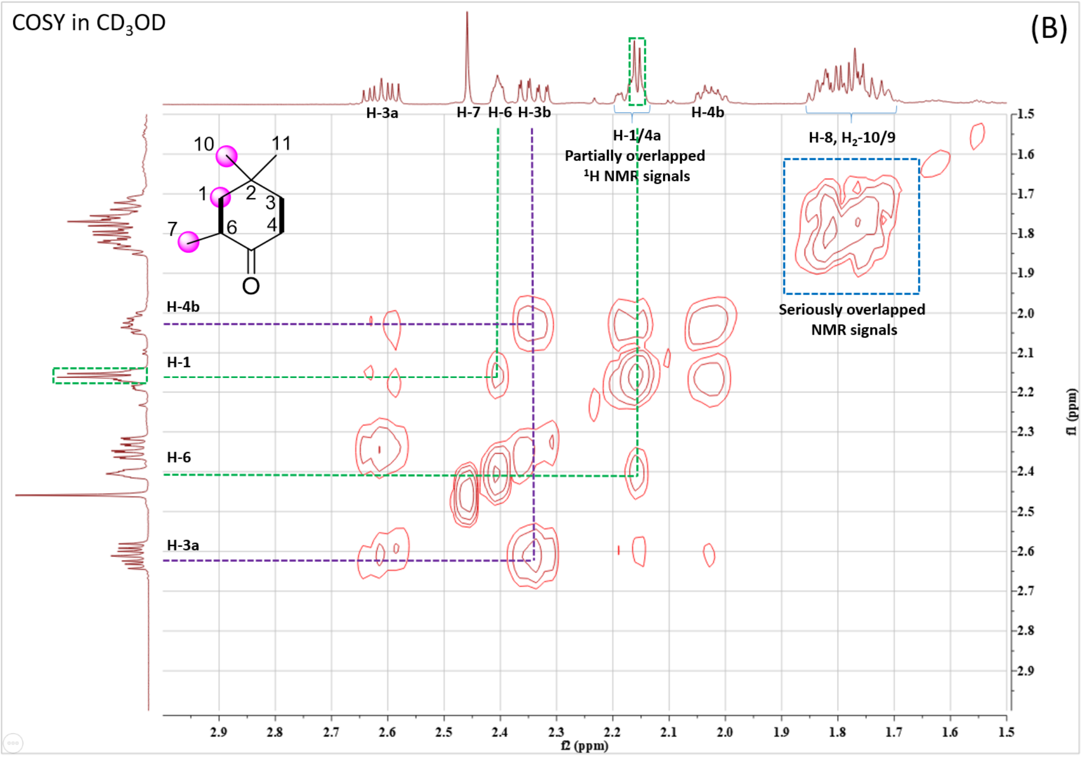

Figure S12. HMBC spectrum of compound **1**.

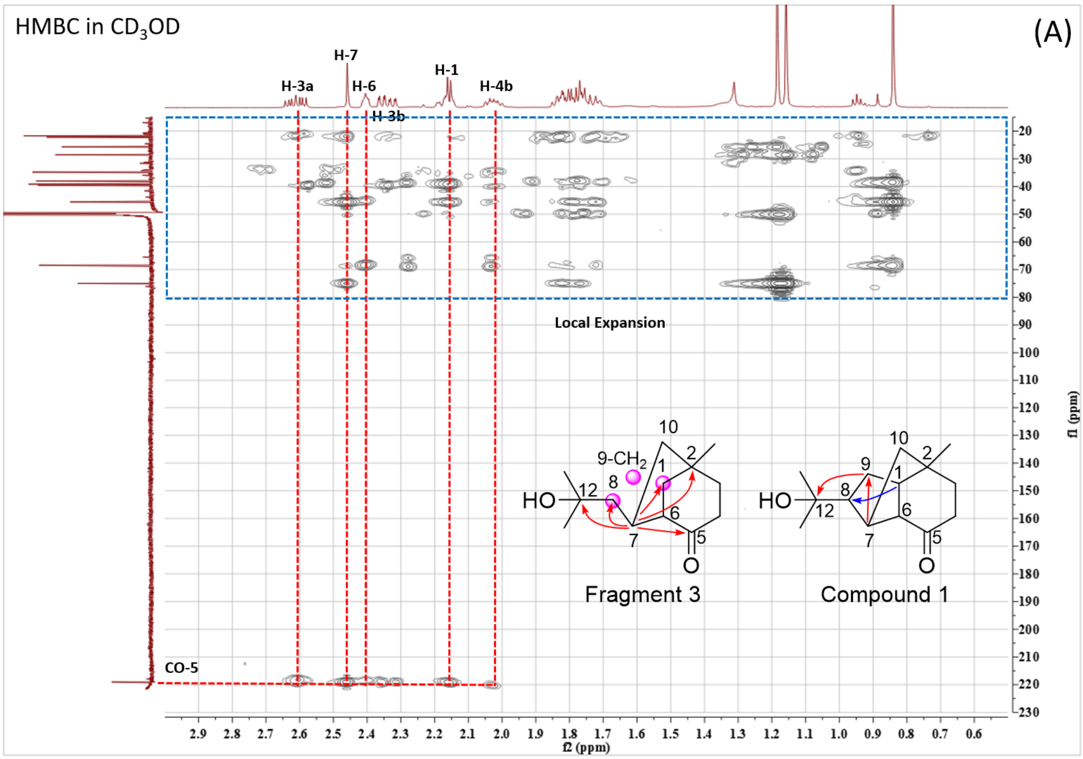

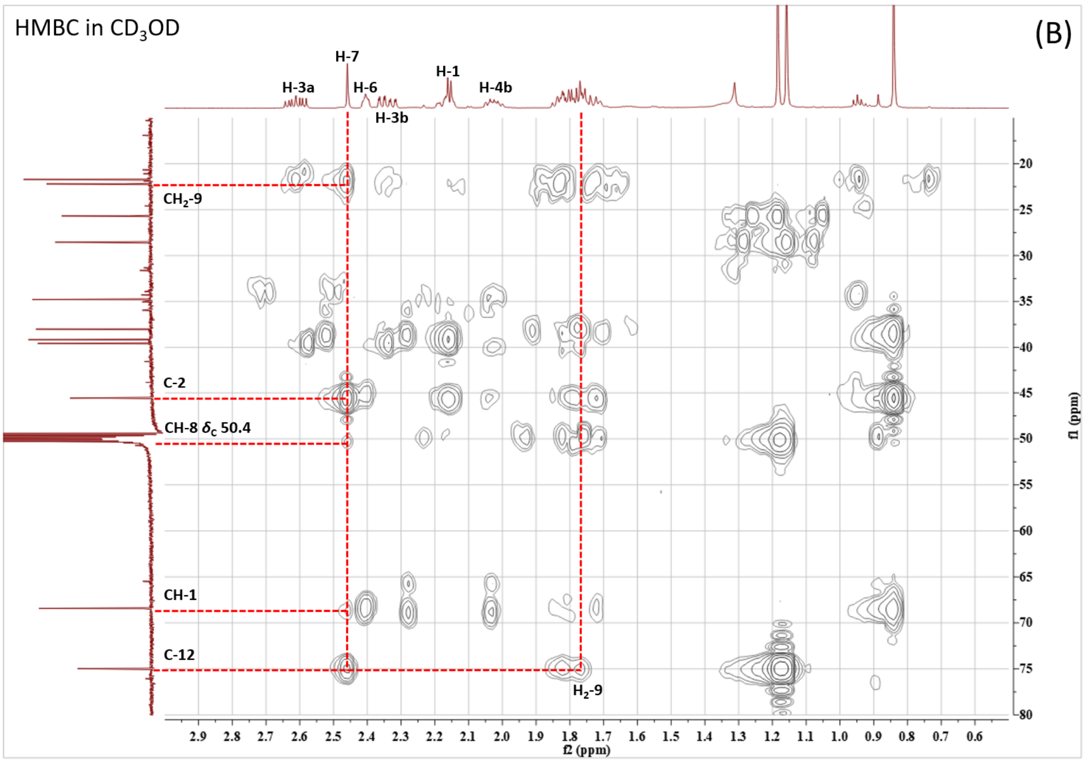

Figure S13. NOESY spectrum of compound **1**.

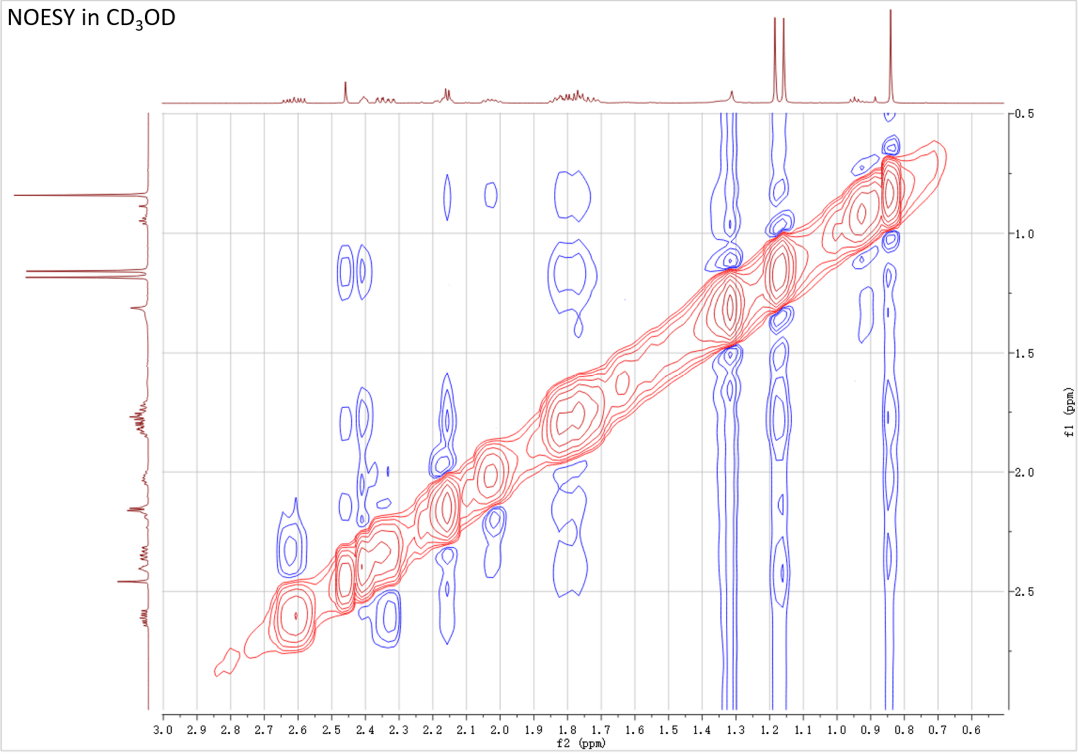

Figure S14. ^{1}H NMR (500 MHz, DMSO-*d*6) spectrum of compound **1**.

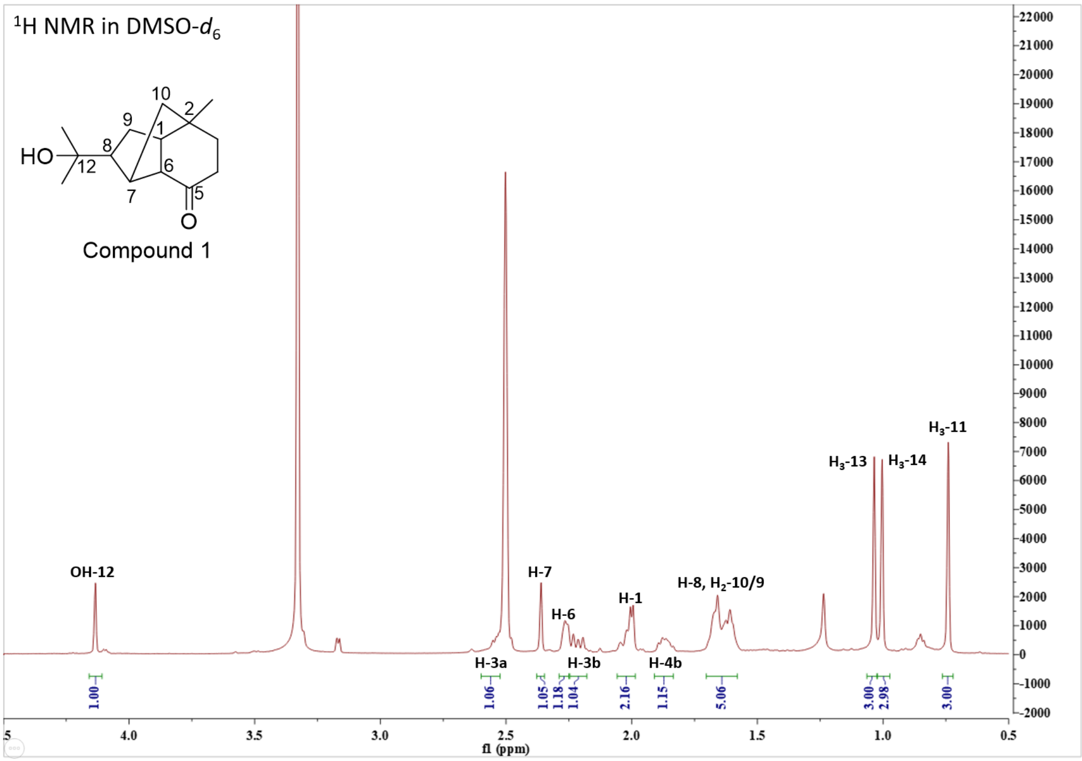

Experimental Section

Method of calculated ECD spectrum of compound 1: Monte Carlo conformational searches were carried out using the Spartan's 10 software using Merck Molecular Force Field (MMFF). The conformers with a Boltzmann population of over 5% were chosen for ECD calculations, and then the conformers were initially optimized at B3LYP/6-31+g (d, p) level in MeOH, using the CPCM polarizable conductor calculation model. The theoretical calculation of ECD was conducted in MeOH using time-dependent density functional theory (TD-DFT) at the B3LYP/6-311+g (d, p) level for all conformers of compound **1**. Rotatory strengths for a total of 50 excited states were calculated. ECD spectra were generated using the program SpecDis 1.6 (University of Würzburg, Würzburg, Germany) and GraphPad Prism 5 (2365 Northside Dr., Suite 560, San Diego, CA 92108) from dipole-length rotational strengths, by applying Gaussian band shapes with sigma = 0.3 eV.
